# Continuous Associations between Remote Self-Administered Cognitive Measures and Imaging Biomarkers of Alzheimer’s Disease

**DOI:** 10.14283/jpad.2024.99

**Published:** 2024-05-29

**Authors:** E. A. Boots, R. D. Frank, W. Z. Fan, T. J. Christianson, W. K. Kremers, J. L. Stricker, M. M. Machulda, J. A. Fields, J. Hassenstab, J. Graff-Radford, P. Vemuri, C. R. Jack, D. S. Knopman, R. C. Petersen, Nikki H. Stricker

**Affiliations:** 1 Divison of Neurocognitive Disorders, Department of Psychiatry and Psychology, Mayo Clinic, Rochester, MN USA; 2 Division of Biomedical Statistics and Informatics, Department of Quantitative Health Sciences, Mayo Clinic, Rochester, Minnesota USA; 3 Department of Information Technology, Mayo Clinic, Rochester, Minnesota USA; 4 Department of Neurology and Psychological & Brain Sciences, Washington University in St. Louis, St. Louis, Missouri USA; 5 Department of Neurology, Mayo Clinic, Rochester, Minnesota USA; 6 Department of Radiology, Mayo Clinic, Rochester, Minnesota USA; 7 ABPP-CN, Mayo Clinic, 200 First Street SW, Rochester, MN 55905 USA

**Keywords:** Digital health, amyloid, mild cognitive impairment, Mayo Test Drive, Neuropsychological Tests

## Abstract

**Background:**

Easily accessible and self-administered cognitive assessments that can aid early detection for Alzheimer’s disease (AD) dementia risk are critical for timely intervention.

**Objectives/Design:**

This cross-sectional study investigated continuous associations between Mayo Test Drive (MTD)–a remote, self-administered, multi-device compatible, web-based cognitive assessment–and AD-related imaging biomarkers.

**Participants/Setting:**

684 adults from the Mayo Clinic Study of Aging and Mayo Clinic Alzheimer’s Disease Research Center participated (age=70.4±11.2, 49.7% female). Participants were predominantly cognitively unimpaired (CU; 94.0%).

**Measurements:**

Participants completed (1) brain amyloid and tau PET scans and MRI scans for hippocampal volume (HV) and white matter hyperintensities (WMH); (2) MTD remotely, consisting of the Stricker Learning Span and Symbols Test which combine into an MTD composite; and (3) in-person neuropsychological assessment including measures to obtain Mayo Preclinical Alzheimer’s disease Cognitive Composite (Mayo-PACC) and Global-z. Multiple regressions adjusted for age, sex, and education queried associations between imaging biomarkers and scores from remote and in-person cognitive measures.

**Results:**

Lower performances on MTD were associated with greater amyloid, entorhinal tau, and global tau PET burden, lower HV, and higher WMH. Mayo-PACC and Global-z were associated with all imaging biomarkers except global tau PET burden. MCI/Dementia participants showed lower performance on all MTD measures compared to CU with large effect sizes (Hedge’s g’s=1.65–2.02), with similar findings for CU versus MCI only (Hedge’s g’s=1.46–1.83).

**Conclusion:**

MTD is associated with continuous measures of AD-related imaging biomarkers, demonstrating ability to detect subtle cognitive change using a brief, remote assessment in predominantly CU individuals and criterion validity for MTD.

**Electronic Supplementary Material:**

Supplementary material is available in the online version of this article at 10.14283/jpad.2024.99.

## Introduction

**N**ovel treatments targeting the underlying pathophysiology of Alzheimer’s disease (AD) are now available (1), with ongoing and future clinical trials likely to result in even more treatment options for individuals with biomarker-confirmed AD (2). Given these new treatment options, along with existing behavioral approaches to prevent or slow the progression of the AD clinical syndrome (3), the need for easily accessible and widespread cognitive screening of older adults is more imperative than ever before as it allows for timely, early detection of cognitive change during a critical time window for treatment intervention. In particular, brief cognitive assessment tools that can be deployed remotely and self-administered have high utility for decentralized clinical trials of AD as well as for use in clinical settings, where these assessments can aid in triage to specialty clinics, inform treatment initiation, support inclusion, and inform cognitive progression (4, 5). Development of brief, digital remote cognitive assessments that detect subtle cognitive change and are sensitive to AD-related pathological change also allow for greater accessibility compared with in-person neuropsychological testing and could allow for future comparison with plasma AD biomarkers, which are themselves poised to become highly accessible when compared with imaging and cerebrospinal fluid biomarkers (6).

Preclinical Alzheimer’s Cognitive Composites (PACCs) (7) have been developed by multiple groups (specific examples include the Alzheimer’s Disease Cooperative Study (ADCS)-PACC (8), the PACC (5, 9) and the Mayo-PACC (10)) and are commonly utilized outcome measures in longitudinal studies and AD clinical trials that have prioritized inclusion of in-person cognitive measures sensitive to subtle cognitive changes observed in preclinical AD. To date, few remote digital assessments have been assessed as measures sensitive to subtle cognitive changes observed in preclinical AD in a comparable fashion to PACCs. One digital assessment, the Computerized Cognitive Composite (C3), showed moderate correlations (r=.39) with PACC (comprised of the Mini Mental Status Exam [MMSE], Wechsler Memory Scales-Revised Logical Memory Delayed Recall, Digit-Symbol Coding Test, and the Free and Cued Selective Reminding Test Free + Total Recall) as well as comparable small cross-sectional group differences across amyloid positive (A+) and amyloid negative (A-) groups (11). However, although capable of being administered remotely, the C3 was completed in person with an administrator present. Another digital assessment that was administered unsupervised, the Boston Remote Assessment for Neurocognitive Health (BRANCH), was strongly correlated with the PACC5 (r=.617) along with cortical amyloid (r=−.205) and entorhinal tau deposition (r=−.178) (12). Additionally, there is evidence to suggest that measures of processing speed/executive function, in addition to memory, may be particularly important to include in composite measures to detect and monitor subtle cognitive changes in individuals with emerging amyloid beta (prior to typical amyloid-positive thresholds) (13). Thus, remote, digital cognitive assessments that employ measures most sensitive to the earliest changes in both cognition and biomarkers of AD could provide a scalable and accessible alternative that is comparable to current person-administered PACCs.

Mayo Test Development through Rapid Iteration, Validation, and Expansion (Mayo Test Drive, MTD) is a remote, self-administered, multi-device compatible, web-based cognitive assessment (14). MTD provides in-depth assessment of key cognitive domains that are sensitive to subtle cognitive changes common in early AD and is specifically designed for remote assessment (15). MTD consists of the Stricker Learning Span (SLS), a novel computer adaptive verbal word list memory test (14–16), as well as the Symbols Test, an open-source measure of processing speed that requires visuospatial discrimination and correlates with measures of processing speed and executive functioning (14). MTD can be easily completed in a remote and self-administered fashion; we have previously shown that MTD has high usability in cohorts of older adults and can typically be completed in about 15 minutes (14, 17). Prior work from our group has also demonstrated that the SLS is able to differentiate between AD biomarker-defined groups comparably to the in-person administered Auditory Verbal Learning Test (AVLT), including distinguishing participants who were A+ versus A− as well as those who were amyloid and tau positive (A+T+) versus amyloid and tau negative (A−T−) (16). Notably, this work was done in a predominantly cognitively unimpaired (CU) sample, suggesting that the SLS can detect subtle objective cognitive decline that aids in differentiation of biomarker positive versus negative participants with no other overt clinical symptoms.

Given that combining measures of learning/memory and processing speed/executive function provides richer information regarding cognitive performance than either in isolation, it is important to understand how performance on a composite measure of MTD is related to biomarkers of AD in a continuous fashion, rather than by splitting into positive and negative groups. Demonstrating that MTD performance is associated with continuous measures of amyloid, tau, neurodegeneration, and vascular injury is in line with the Alzheimer’s Association research framework for biological diagnosis of AD (18) (currently under revision), as well. Currently, most PACCs rely on z-scores that are study specific, where z-scores are typically referenced to the sample presented within the study, making interpretation of performance across studies more challenging (10, 19, 20). In the current study we introduce an MTD raw composite score that can be interpreted independent of any specific study sample. Additionally, a raw score composite can facilitate future development of easily understood and generalizable cut-offs that do not adjust away the effects of age, which is confounded with increased prevalence of AD biomarkers and other undetected neuropathologies (21, 22) and thereby decreases sensitivity to mild cognitive impairment (MCI) (23). This MTD raw composite thus retains the benefits of a total raw score, which can be used for screening purposes, and can also be used in conjunction with normative data (24, 25).

The primary aim of this study was to investigate continuous associations between MTD and imaging biomarkers to examine the criterion validity of MTD and its subtests as a measure of AD-related cognitive change in a population-based study of aging. Specifically, we hypothesized that worse performances on MTD would be associated with greater amyloid and tau PET burden, lower hippocampal volumes (HV), and higher white matter hyperintensity (WMH) volume. A secondary aim of this study was to validate a new MTD raw composite variable. We hypothesized that the MTD raw composite variable would demonstrate a similar pattern of results compared to both an alternative MTD composite that uses a traditional z-score approach and to existing in-person cognitive composite measures in association with imaging biomarkers. Finally, we also report group difference effect sizes for MTD composite and subtest variables across diagnostic groups (CU, MCI, and dementia) to inform potential clinical utility and validity, as well as correlations between MTD and imaging biomarkers across diagnostic groups.

## Methods

### Participants

The majority of participants (98.5%) were from the Mayo Clinic Study of Aging (MCSA). The MCSA is a longitudinal, population-based study of aging for residents of Olmsted County, Minnesota. Participants are recruited via random sampling of age- and sex-stratified groups using the Rochester Epidemiology Project medical records-linkage system (26). Individuals with dementia or who are terminally ill or in hospice are not eligible for MCSA study enrollment. Once enrolled, however, individuals can remain in the study if they later convert to dementia. Because of this population-based sampling design, most participants in the MCSA are cognitive unimpaired. Study visits involve a physician examination, study coordinator interview that includes the Clinical Dementia Rating (CDR®) instrument (27), and neuropsychological testing (28). After each study visit, a diagnosis of CU, MCI (29), or dementia (30) is established after the examining physician, interviewing study coordinator, and neuropsychologist make independent diagnostic determinations and come to consensus agreement (28). All participants are also invited to undergo and sign informed consent for neuroimaging. Participants with MCI or dementia complete study visits every 15 months, whereas CU individuals complete visits every 30 months if age 50 or older, or every 60 months if younger than 50 years of age. The remainder of participants were recruited from the Mayo Clinic Alzheimer’s Disease Research Center (ADRC; Rochester, MN). This study was approved by the Mayo Clinic and Olmsted Medical Center Institutional Review Boards and conducted in accordance with the Declaration of Helsinki. Written consent was obtained from all participants for participation in the overall study protocols (MCSA or ADRC) and oral consent was obtained for participation in the ancillary study protocol by which MTD was obtained via remote data collection.

Inclusion criteria for this specific study consisted of completion of MTD and available neuroimaging data (MRI and/or PET) completed within 36 months of completion of MTD. Participants were included across all diagnostic categories (i.e., CU, MCI, and dementia). Most participants had both MRI and PET neuroimaging data available as well as complete in-person neuropsychological data; however, specific sample sizes varied across outcome measures (see Table 1).

**Table 1:** Participant Characteristics for the Full Sample as well as for Cognitively Unimpaired and Mild Cognitive Impairment/Dementia Participants

Characteristic*	**Full Sample N=684**^**1**^	**CU N=643**	**MCI/Dementia N=41**	p†	**Hedge’s g**
Demographics					
Age at MCSA/ADRC visit, years	70.363 (11.204)	69.890 (11.111)	77.786 (10.097)	<0.001	0.71 (0.40, 1.03)
Sex, N (%) Female	340 (49.7%)	318 (49.5%)	22 (53.7%)	0.60 ‡	—
Education, years	15.646 (2.381)	15.719 (2.335)	14.512 (2.812)	0.002	−0.51 (−0.83, −0.19)
Race, N (%) White^2^	664 (97.1%)	624 (97.0%)	40 (97.6%)	1.00 §	—
Ethnicity, N (%) Non-Hispanic^3^	680 (99.4%)	639 (99.4%)	41 (100.0%)	1.00 §	—
In-person visit to MTD, months	0.737 (1.965)	0.709 (1.896)	1.169 (2.831)	0.15	0.23 (−0.08, 0.55)
Imaging to MTD, months	5.746 (11.393)	6.113 (11.622)	−0.016 (3.492)^4^	<0.001	−0.54 (−0.86, −0.23)
MTD completed in clinic, N (%)	3 (0.4%)	3 (0.5%)	0 (0.0%)	1.00 §	—
Cognition: Mayo Test Drive^5,7^					
MTD-SBCr, n=680	105.221 (22.675)	107.653 (20.171)	66.314 (25.200)	<0.001	−2.02 (−2.35, −1.68)
MTD-SBCz, n=680	−0.121 (1.124)	0.000 (1.000)	−1.830 (1.057)	<0.001	−2.10 (−2.44, −1.76)
SLS Sum of Trials, n=681	74.023 (18.747)	75.833 (17.174)	45.025 (19.265)	<0.001	−1.78 (−2.11, −1.45)
SYM, n=681	3.472 (1.313)	3.337 (1.067)	5.623 (2.513)	<0.001	1.91 (1.57, 2.24)
SYMaw, n=681	31.219 (6.849)	31.839 (6.065)	21.289 (10.338)	<0.001	−1.65 (−1.98, −1.32)
Cognition: In-Person Measures^6,7^					
Mayo-PACC z, n=661	−0.123 (1.144)	0.000 (1.000)	−2.356 (1.368)	<0.001	−2.28 (−2.64, −1.92)
Global Cognition z, n=629	−0.111 (1.132)	0.000 (1.000)	−2.694 (0.923)	<0.001	−2.70 (−3.12, −2.28)
AVLT Sum of Trials, n=668	65.177 (18.759)	66.979 (17.568)	35.289 (11.130)	<0.001	−1.83 (−2.18, −1.49)
Trail Making Test B, n=669	77.166 (43.657)	72.472 (34.875)	157.351 (83.977)	<0.001	2.17 (1.82, 2.52)
Digit Symbol Coding, n=658	51.299 (12.650)	52.060 (12.222)	34.179 (9.843)	<0.001	−1.47 (−1.86, −1.09)
STMS, n=677	35.581 (2.624)	35.962 (1.905)	29.500 (4.449)	<0.001	−3.02 (−3.38, −2.67)
Neuroimaging Metrics^5^					
Amyloid PET Meta-ROI SUVR, n=670	1.549 (0.375)	1.527 (0.341)	1.890 (0.637)	<0.001	0.99 (0.67, 1.32)
Tau PET Meta-ROI SUVR, n=667	1.209 (0.140)	1.197 (0.098)	1.389 (0.374)	<0.001	1.45 (1.13, 1.78)
Tau PET EC-ROI SUVR, n=667	1.134 (0.159)	1.120 (0.130)	1.360 (0.322)	<0.001	1.62 (1.29, 1.95)
Hippocampal Volume z-score, n=680	−0.343 (0.686)	−0.283 (0.611)	−1.292 (1.023)	<0.001	−1.57 (−1.90, −1.24)
% WMH Volume, ln, n=665	−0.645 (0.904)	−0.680 (0.884)	−0.111 (1.047)	<0.001	0.64 (0.32, 0.95)

*Values are presented as Mean (Standard Deviation) unless otherwise noted; † All between-group comparisons are T-tests unless otherwise indicated; ‡ Chi-square; §Fisher; 1. n=674 Mayo Clinic Study of Aging (MCSA); n=10 Mayo Alzheimer’s Disease Research Center (ADRC) Rochester, MN; 2. n=7 Asian, n=5 Black, n=8 Missing/Unknown; 3. n=2 Missing/Unknown; 4. Negative value indicates this group on average had imaging visits prior to MTD completion. By study design, individuals with MCI/dementia complete imaging every 15 months, whereas CU individuals complete imaging every 30 months if age 50 or older, or every 60 months if younger than 50 years of age; 5. Mayo Test Drive and Neuroimaging are independent of diagnosis (data not considered for consensus diagnosis); 6. Results of in-person cognitive measures are considered for consensus diagnosis; 7. Results remained significant when adjusting for age, sex, and education (all p’s≤.003). Note: ADRC = Alzheimer’s Disease Research Center; AVLT = Auditory Verbal Learning Test; CU = Cognitively Unimpaired; EC = entorhinal cortex; Mayo-PACC = Mayo Preclinical Alzheimer’s Disease Cognitive Composite; MCI = Mild Cognitive Impairment; MCSA=Mayo Clinic Study of Aging; MRI = Magnetic Resonance Imaging; MTD = Mayo Test Drive; MTD-SBCr = Mayo Test Drive Screening Battery Composite raw; MTD-SBCz = Mayo Test Drive Screening Battery Composite z; p = p-value; PET = Positron Emission Tomography; ROI = Region of Interest; SLS = Stricker Learning Span; STMS = Short Test of Mental Status; SUVR = Standard Uptake Volume Ratio; SYM = Symbols Test average correct item response time; SYMaw= Symbols Test accuracy-weighted average correct item response time; WMH = White Matter Hyperintensities.

### Self-Administered, Remote Cognitive Measures

All participants completed MTD (14, 16) approximately a few weeks following their in-person MCSA or ADRC visit (0.74 months on average). Participants were invited via email to complete the measures remotely, and nearly all participants completed MTD remotely and unsupervised. MTD is comprised of the SLS and the Symbols Test, from which composite measures are calculated, as described below.

#### Stricker Learning Span (SLS)

The SLS is a computer adaptive list learning task; please see prior publications for a detailed review of test procedures (14–16). Briefly, single words are visually presented sequentially across five learning trials. After each list presentation, memory for each word on the word list is assessed with four-choice recognition. The SLS begins with eight items and then the number of words either stays the same, increases, or decreases depending on performance across each trial (range 2–23 words by trial five). The delay trial of the SLS occurs following completion of the Symbols Test and presents all items that were previously presented on any SLS learning trial. The SLS Sum of Trials, the primary outcome variable from this task, is calculated as the sum of words correctly remembered from Trials 1–5 and the Delay trial. Additional secondary SLS variables include SLS Maximum Learning Span, SLS Trials 1–5 Total, and SLS Delay.

#### Symbols Test

The Symbols Test is an open-source measure of processing speed with previously demonstrated validity and reliability (31, 32). Processing speed measures are routinely incorporated into composite cognitive measures designed to be sensitive to early preclinical changes due to their known sensitivity to cognitive aging, AD, and other neurodegenerative disorders (14, 33). For each item, participants identify which of two symbol pairs on the bottom of the screen matches one of three symbol pairs presented at the top of the screen. The original version is part of the Ambulatory Research and Cognition (ARC) app (34) and includes up to 28 12-item trials taken over the course of seven consecutive days. The number of trials for this shortened version (four 12-item sequential trials for a total of 48 items) was selected based on data showing that between-person reliability passes a 0.80 reliability threshold after 2 trials (32). The primary outcome variable used in other studies is average correct item response time in seconds across trials (SYM). Although the primary outcome variable only includes correct items, we have observed that response times tend to be consistent, whether accurate or inaccurate. Clinically, there may also be some utility in understanding how accuracy influences performance. For this reason, we created a modification of the SYM variable that additionally accounts for accuracy, SYMaw, which is defined below.

#### MTD Composite Measures

We included two MTD composite variables in this study. The first, MTD screening battery composite z (MTD-SBCz), is a composite based on a traditional z-score approach that previously demonstrated good diagnostic accuracy for MCI in preliminary data (35). This composite is the average of the z-scores of the following four variables, which were created from all CU participants in this sample: SLS Maximum Learning Span, SLS Trials 1–5 Total, SLS Delay, and SYM (SYM is multiplied by −1 to invert such that higher scores reflect better performance). We then used the mean and standard deviation of just the CU population to z-score the entire sample for analysis.

The second MTD composite variable is the MTD screening battery composite raw (MTD-SBCr). For this composite, we aimed to create a composite on a raw score scale that performed similarly to the MTD-SBCz but also factored in accuracy on the Symbols test, in addition to speed, to help capture variance observed in participants with low accuracy. Given that accuracy on the Symbols test has a skewed distribution with most participants performing at ceiling (i.e., 48/48 items correct), we assigned participants an accuracy weighting score (1–5) based on their accuracy performance as follows: 1 = <35 correct; 2 = 35–41 correct; 3 = 42 correct; 4 = 43 correct; 4.25 = 44 correct; 4.5 = 45 correct; 4.75 = 46 correct; 5 = 47–48 correct. Next, we subtracted SYM from 10 seconds to inverse the variable such that higher scores indicated better performance (in the original variable, faster, i.e., lower, scores indicate better performance) in order to combine the variable with SLS performance for the composite. We selected 10 seconds as the time to subtract from because nearly all participants had an average correct items response time of less than 10 seconds when this composite was a priori conceptualized (April 30, 2022). Then, we multiplied the rescaled SYM variable by the accuracy-weighting score (1–5 defined above) to compute an accuracy-weighted average correct item response time variable (SYMaw). For the few participants for whom SYM was >10 seconds, participants received 0 on the SYMaw variable. Thus, the equation for SYMaw is as follows, where SYM = Symbols average correct items response time in seconds: SYMaw = (10 – SYM) * accuracy-weighting score. Finally, to complete calculation of the MTD-SBCr, we added SLS Sum of Trials and the SYMaw variable. MTD-SBCz and MTD-SBCr are both more heavily weighted toward SLS performance. As expected, MTD-SBCz and MTD-SBCr are highly correlated (r=0.98). See Supplemental Online Resources for additional details about these computations and the relative weighting of the composites.

### In-Person Cognitive Measures

All participants completed an in-person neuropsychological evaluation that was administered by a psychometrist and supervised by a board-certified neuropsychologist (JAF or MMM). The evaluation includes nine neuropsychological tests that together comprise four cognitive domains. The cognitive domains and their constituent tests are as follows: Memory – Auditory Verbal Learning Test (AVLT) delayed recall (36) and Wechsler Memory Scale-Revised Logical Memory II & Visual Reproduction II (37); Language – Boston Naming Test (38) and Category Fluency (39); Executive Function – Trail Making Test B (40) and Wechsler Adult Intelligence Scale-Revised (WAIS-R) Digit Symbol Coding subtest (41); and Visuospatial Skills – WAIS-R Picture Completion and Block Design subtests (41). Given interest in comparing MTD cognitive outcomes with comparable in-person neuropsychological measures, we only included performances on the AVLT, Trail Making Test B, and WAIS-R Digit Symbol Coding in our subtest analyses.

Additionally, two in-person composite cognitive measures were calculated from the above neuropsychological test data. First, the Mayo Clinic Preclinical Alzheimer’s Cognitive Composite (Mayo-PACC) is an average of z-scores from the AVLT sum of trials, Trail Making Test B (reversed) and animal fluency (10). Second, a Global Cognition z-score was calculated as the average of z-scores from all nine neuropsychological tests; this is routinely used in MCSA publications (see Stricker et al. (10) for comparison to several PACCs). Like for MTD-SBCz, both Mayo-PACC and Global Cognition z-score composites were calculated using CU participants from this study sample as the reference group. Finally, the Short Test of Mental Status (STMS) is a brief, multi-domain cognitive screening measure (38 points possible) that was administered to all participants by the study physician (42). The STMS has been shown to have greater ability to detect MCI relative to the MMSE (43) and the MoCA (44) and is included for reference.

### Neuroimaging Acquisition & Processing

#### Amyloid PET

Amyloid PET scans were acquired via a GE Discovery RX or DXT PET/CT scanner using the 11C-Pittsburgh Compound B (PiB) ligand as previously described (45). A PiB PET standardized uptake value ratio (SUVR) was defined for each participant by computing the median PiB uptake in gray and white matter of the prefrontal, orbitofrontal, parietal, temporal, anterior cingulate, and posterior cingulate/precuneus regions of interest (ROI) and dividing this by the median uptake in the cerebellar crus gray matter. Together, this continuous SUVR metric is referred to as the amyloid PET meta-ROI and was our measure of interest for amyloid burden. This measure was natural log transformed and z-scored for analyses to facilitate comparisons of results.

#### Tau PET

Tau PET scans were also acquired using a GE Discovery RX or DXT PET/CT scanner using the flortaucipir (18F-AV-1451) ligand as previously described (46, 47). Two tau PET metrics were used for the purposes of this study. First, flortaucipir PET SUVR was calculated using the median uptake in gray and white matter of the entorhinal, amygdala, parahippocampal, fusiform, inferior temporal, and middle temporal ROIs divided by the median uptake in the cerebellar crus gray matter; this metric is referred to as the tau PET meta-ROI as a more global measure of tau burden. Second, given previous associations between flortaucipir uptake in the entorhinal cortex (EC) and PiB in CU participants in our sample (48), we also investigated this region individually, which is referred to as Tau PET EC-ROI. Both SUVR metrics were investigated continuously and were natural log transformed and z-scored for analysis.

#### Hippocampal Volume

MRI scans were conducted on a Siemens 3T Prisma scanner using a 3D Magnetization Prepared Rapid Acquisition Gradient-Echo (MPRAGE) sequence. As previously described (14, 49), SPM12 Unified Segmentation was used for tissue-class segmentation with Mayo Clinic Adult Lifespan Template (MCALT; https://www.nitrc.org/projects/mcalt/) settings optimized for the study population. Advanced Normalization Tools (ANTs) symmetric normalization was used to warp the MCALT-ADIR122 atlas for computing intracranial volume (ICV) as well as HV (50, 51). To adjust HV for ICV, residual values from the linear regression of ICV (x) and HV (y) were calculated with sex-specific formulas as previously described (14). Values were additionally natural log transformed and z-scored.

#### White Matter Hyperintensities

For quantifying WMH, 3D T2-weighted fluid attenuated inversion recovery (FLAIR) MRI sequences were acquired and co-registered to MPRAGE images (52, 53). WMH was divided by ICV and multiplied to create a percentage WMH volume, which was natural log transformed due to data skewness and then z-scored.

### Statistical Analyses

Analyses were conducted in SAS version 9.4 (SAS Institute Inc., Cary, NC), with a two-tailed p-value of ≤.05 for significance. Demographics and other participant characteristics for the full sample and by diagnostic categories were summarized using means and standard deviations for continuous data and by counts and percentages for categorical data. Basic between-group differences were calculated using independent-samples t-test for continuous variables and *χ*2 or Fisher tests for categorical variables. Linear regression was used to additionally adjust for age, sex, and education for between-group differences by clinical diagnosis for cognitive data. Hedge’s g was used to calculate effect sizes for between-group differences of continuous data.

For primary analyses, multivariable linear regression was used to investigate associations between five neuroimaging biomarkers, including amyloid PET meta-ROI, tau PET meta-ROI, tau PET EC-ROI SUVRs, HV, and percentage WMH volume (analyzed separately), and remote (MTD-SBCr and MTD-SBCz) and in-person (Mayo-PACC, Global z, STMS) composite indices of cognition while adjusting for age, sex, and education. To visually compare the mean estimates from each linear regression model, we opted to z-score these outcomes, with the CU sample as the referent group. Multivariable linear regression was also used to analyze associations between the five neuroimaging biomarkers (analyzed separately) and remote and in-person cognitive measures of memory (SLS Sum of Trials, SLS Maximum Learning Span, SLS Trials 1–5 Total, SLS Delay and AVLT Sum of Trials) and processing speed/executive function (SYM, SYMaw, Trail Making Test B, and Digit Symbol Coding).

Finally, Spearman correlation coefficients were utilized to examine unadjusted associations between imaging biomarkers and remote and in-person cognitive measures, both within the full sample and within diagnostic category.

## Results

### Participant Characteristics

Based on study inclusion criteria, 684 individuals comprised the study sample. The average age at completion of MTD was 70.36 years (11.20 SD), and average time from neuroimaging visit to completion of MTD was 5.75 months (11.39 SD). Approximately 50.3% of the sample was male and participants had about 16 years of education on average. Of study participants, 643 were CU (94%), 34 were diagnosed with MCI (5%); and 7 were diagnosed with dementia (1%) based on consensus conference. Given the small number of participants with dementia, MCI and dementia diagnostic groups were combined in Table 1. There were differences between diagnostic groups in terms of age (p<.001) and education (p=.002). Nearly all participants completed MTD remotely (99.6%), with a small number of participants electing to complete MTD in clinic (n=3). In terms of device use for completing MTD, 63.5% of participants used a desktop computer or laptop, 21.4% used a smartphone, and 14.8% used a tablet (0.3% device is unknown). For additional participant details, see Table 1. Potential interference (during any subtest) was endorsed on questions embedded into each MTD session by 16.8% of participants and noise was endorsed during the session by 4.5% of participants. Five (0.7%) participants endorsed potential task interference due to technical problems.

Spearman correlation coefficients between remote and in-person cognitive tests showed significant associations (all p’s < .001; see Table S1 for full details). Primary associations of interest were robust including associations between MTD-SBCr and other global measures of cognition (Mayo-PACC r=0.68; Global-z r=0.67; Kokmen STMS r=0.56), the SLS and AVLT Sum of Trials (r=0.61), and SYMaw and other measures of processing speed/executive function (Trails B r=−.61; Digit Symbol Coding r=0.59).

### Associations of Neuroimaging Biomarkers and Remote and In-Person Cognitive Measures

#### Composite Cognitive Measures

In models adjusted for age, sex, and education, all neuroimaging biomarkers (amyloid meta-ROI, tau meta-ROI, tau EC-ROI, HV, and WMH volume) were significantly associated with remote measures including the MTD-SBCr and MTD-SBCz (all p’s<.001). When investigating similar associations with in-person cognitive measures, the amyloid meta-ROI (p’s≤.03), tau EC-ROI (p’s≤.03), HV (p’s<.001), and WMH volume (p’s<.001), but not tau meta-ROI (p’s≥.10), were associated with both Mayo-PACC and Global Cognition Z. All neuroimaging biomarkers were significantly associated with the STMS (p’s≤.03). For additional details, see Table 2.

**Table 2:** Association Between Standardized Neuroimaging Biomarkers and Composite Cognitive Outcomes Adjusting for Age, Sex, and Education using Linear Regression Models

	**Amyloid PET Meta-ROI SUVR**		**Tau PET Meta-ROI SUVR**		**Tau PET EC-ROI SUVR**		**Hippocampal Volume z-score**		**% WMH Volume, ln**	
**Cognitive Measure**	**Mean estimate (95% CI)**	**p**	**Mean estimate (95% CI)**	**p**	**Mean estimate (95% CI)**	**p**	**Mean estimate (95% CI)**	**p**	**Mean estimate (95% CI)**	**p**
Remote										
MTD-SBCr	−0.15 (−0.23, −0.08)	<.001	−0.19 (−0.25, −0.13)	<.001	−0.24 (−0.30, −0.17)	<.001	0.24 (0.17, 0.31)	<.001	−0.19 (−0.28, −0.09)	<.001
MTD-SBCz	−0.16 (−0.24, −0.09)	<.001	−0.20 (−0.26, −0.13)	<.001	−0.24 (−0.31, −0.18)	<.001	0.25 (0.18, 0.32)	<.001	−0.19 (−0.28, −0.09)	<.001
In-Person										
Mayo-PACC	−0.14 (−0.21, −0.06)	<.001	−0.05 (−0.12, 0.01)	0.10	−0.10 (−0.16, −0.03)	0.005	0.20 (0.13, 0.27)	<.001	−0.17 (−0.26, −0.08)	<.001
Global Cognition Z	−0.09 (−0.16, −0.01)	0.03	−0.00 (−0.07, 0.07)	0.99	−0.08 (−0.15, −0.01)	0.03	0.21 (0.14, 0.28)	<.001	−0.15 (−0.24, −0.06)	<.001
STMS	−0.11 (−0.21, −0.01)	0.03	−0.23 (−0.31, −0.15)	<.001	−0.23 (−0.32, −0.14)	<.001	0.30 (0.21, 0.40)	<.001	−0.18 (−0.30, −0.06)	0.004

Note: All cognitive measures and imaging biomarkers were additionally z-scored to facilitate estimate comparisons within the table. CI = confidence interval; EC = entorhinal cortex; Global Cognition z = average z across all neuropsychological tests administered during an in-person visit; Mayo-PACC = Mayo Preclinical Alzheimer’s disease Cognitive Composite (average z of Auditory Verbal Learning Test sum of trials, animal fluency, and inversed Trails B); MTD = Mayo Test Drive; MTD-SBCr = Mayo Test Drive Screening Battery Composite raw (Stricker Learning Span sum of trials + Symbols Test accuracy-weighted average correct items response time); MTD-SBCz = Mayo Test Drive Screening Battery Composite z; p = p-value; PET = Positron Emission Tomography; ROI = region of interest; STMS = Short Test of Mental Status, which is similar to the MMSE, is included for reference; SUVR = Standard Uptake Volume Ratio; WMH = white matter hyperintensities.

#### Individual Cognitive Measures

In investigating the relationship between neuroimaging biomarkers and memory measures, all neuroimaging biomarkers were associated with the remote measure, SLS Sum of Trials (p’s≤.002). All biomarkers were also associated with the in-person measure, AVLT Sum of Trials (p’s≤.004).

For measures of processing speed/executive function, all neuroimaging biomarkers were significantly associated with both SYM and SYMaw (p’s≤.02). For in-person measures, all neuroimaging biomarkers were associated with Trail Making Test B performance (p’s≤.003). Only HV and WMH volume were associated with Digit Symbol Coding (p’s≤.009). For additional details, see Table 3.

**Table 3:** Association Between Standardized Neuroimaging Biomarkers and Cognitive Subtest Outcomes Adjusting for Age, Sex, and Education using Linear Regression Models

	**Amyloid PET Meta-ROI SUVR**		**Tau PET Meta-ROI SUVR**		**Tau PET EC-ROI SUVR**		**Hippocampal Volume z-score**		**% WMH Volume, ln**	
**Memory Measures**	**Mean estimate (95% CI)**	**p**	**Mean estimate (95% CI)**	**p**	**Mean estimate (95% CI)**	**p**	**Mean estimate (95% CI)**	**p**	**Mean estimate (95% CI)**	**p**
*Remote*										
SLS Sum of Trials	−0.12 (−0.20, −0.05)	0.002	−0.19 (−0.25, −0.13)	<.001	−0.24 (−0.30, −0.17)	<.001	0.21 (0.14, 0.29)	<.001	−0.18 (−0.28, −0.09)	<.001
SLS Max Span	−0.12 (−0.20, −0.05)	0.002	−0.19 (−0.25, −0.13)	<.001	−0.23 (−0.30, −0.17)	<.001	0.21 (0.14, 0.28)	<.001	−0.17 (−0.26, −0.07)	<.001
SLS Trial 1–5 Total	−0.12 (−0.20, −0.05)	0.002	−0.18 (−0.24, −0.12)	<.001	−0.22 (−0.29, −0.16)	<.001	0.21 (0.14, 0.28)	<.001	−0.19 (−0.28, −0.10)	<.001
SLS Delay	−0.10 (−0.18, −0.03)	0.007	−0.18 (−0.24, −0.12)	<.001	−0.24 (−0.30, −0.17)	<.001	0.20 (0.12, 0.27)	<.001	−0.13 (−0.23, −0.04)	0.006
*In-Person*										
AVLT Sum of Trials	−0.14 (−0.21, −0.06)	<.001	−0.10 (−0.16, −0.04)	0.001	−0.14 (−0.20, −0.08)	<.001	0.18 (0.11, 0.25)	<.001	−0.13 (−0.22, −0.04)	0.004
Processing Speed/Executive Function Measures										
*Remote*										
SYM	0.18 (0.09, 0.26)	<.001	0.12 (0.05, 0.19)	<.001	0.13 (0.06, 0.21)	<.001	−0.23 (−0.31, −0.15)	<.001	0.15 (0.05, 0.26)	0.005
SYMaw	−0.14 (−0.22, −0.07)	<.001	−0.12 (−0.18, −0.06)	<.001	−0.13 (−0.19, -0.06)	<.001	0.20 (0.13, 0.27)	<.001	−0.11 (−0.21, −0.01)	0.02
*In-Person*										
Trail Making Test B	0.21 (0.13, 0.30)	<.001	0.13 (0.06, 0.20)	<.001	0.12 (0.04, 0.19)	0.003	−0.20 (−0.28, −0.11)	<.001	0.17 (0.06, 0.27)	0.002
Digit Symbol Coding	−0.04 (−0.11, 0.03)	0.27	0.02 (−0.04, 0.08)	0.56	0.01 (−0.05, 0.07)	0.82	0.09 (0.02, 0.15)	0.008	−0.11 (−0.18, −0.03)	0.009

Note: All cognitive measures and imaging biomarkers were additionally z-scored to facilitate comparisons within the table. AVLT = Auditory Verbal Learning Test; AVLT Sum of Trials = AVLT 1–5 total + Trial 6 + 30-minute delay; CI = confidence interval; Digit Symbol Coding = WAIS-R Digit Symbol Substitution Test; EC = entorhinal cortex; p = p-value; PET = Positron Emission Tomography; ROI = region of interest; SLS = Stricker Learning Span; SLS Sum of Trials = SLS 1–5 total + delay; SLS Max = Maximum learning span achieved during SLS trials 1–5; SUVR = Standard Uptake Volume Ratio; SYM = Symbols Test average correct item response time; SYMaw=Symbols Test accuracy-weighted average correct item response time; Trail Making Test B = Trail Making Test B completion time; WMH = white matter hyperintensities.

### Comparing MTD Performances Between Diagnostic Groups

As anticipated, differences were observed in MTD performance by diagnostic category. Specifically, participants in the MCI/dementia category demonstrated worse performances across all MTD measures relative to participants in the CU category (all p’s<.001), with large effect sizes for both MTD composites (Hedges g=−2.02 and −2.10), the memory measure, SLS Sum of Trials (Hedges g=−1.78), and processing speed/executive function measures including both Symbols measures (Hedges g=−1.65 and 1.91). Group differences remained significant after adjusting for age, sex, and education (all p’s−.003; see Table 1). Supplementary analyses (see Table S2) show that these findings are similar when limited to participants in the CU versus MCI categories, with all p’s<.001 and large effect sizes for MTD composite measures (Hedges g=−1.83 and −1.90), SLS Sum of Trials (Hedges g=−1.62), and for both variants of the Symbols measure (Hedges g=−1.46 and 1.69). Although results must be viewed cautiously given the small number of dementia participants (N=7), preliminary findings comparing MTD performances between those diagnosed with MCI versus dementia suggest a further decrease in performance with increased disease severity. Those diagnosed with dementia showed lower MTD performances for the MTD composite measures and SLS Sum of Trials (p’s≤.03; Hedges g=−0.94 to −1.08); Symbols measures approached significance (p’s=.06; Hedges g =−0.81 to 0.82). See Table S2 for additional details.

### Correlations between Imaging Biomarkers and Remote and In-Person Cognitive Tests by Diagnostic Category

**Figure 1:**
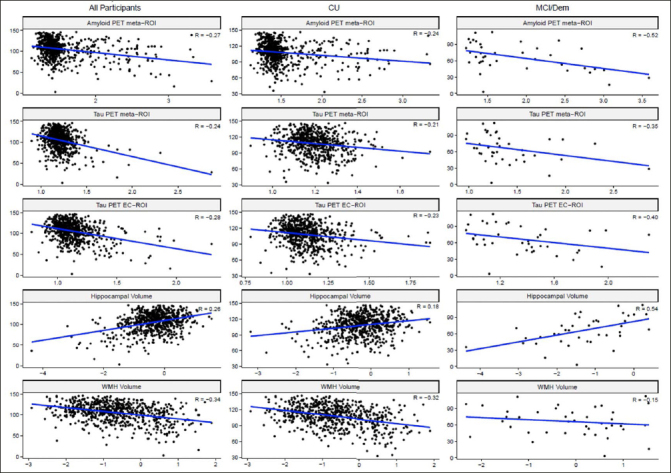
Scatterplots depicting associations between the MTD raw screening battery composite (MTD-SBCr) score (y-axis) and each imaging variable (x-axis) for the full sample (left panel), in cognitively unimpaired participants (center panel), and in participants with MCI or dementia (right panel) Note: Hippocampal volume values are z-scored and WMH volume is a percentage that has been natural log-transformed as outlined in the Methods section. CU = Cognitively Unimpaired; Dem = Dementia; EC = entorhinal cortex; MCI = Mild Cognitive Impairment; MTD-SBCr = Mayo Test Drive Screening Battery Composite raw score; PET = Positron Emission Tomography; ROI = region of interest; WMH = white matter hyperintensities.

Examination of Spearman correlation coefficients between imaging biomarkers and cognitive measures revealed that for individuals who were diagnosed with either MCI or dementia, correlations were generally stronger when compared with CU participants for both remote and in-person cognitive measures. Please see Table 4 and Figure 1 for additional details. For correlations between imaging biomarkers and cognitive measures in the full sample, please see Table S3.

**Table 4:** Spearman Correlations between Neuroimaging Metrics and Cognitive Measures by Diagnosis

	**Amyloid PET Meta-ROI SUVR**		**Tau PET Meta-ROI SUVR**		**Tau PET EC-ROI SUVR**		**Hippocampal Volume z-score**		**% WMH Volume, ln**	
	**CU**	**MCI/DEM**	**CU**	**MCI/DEM**	**CU**	**MCI/**	**CU**	**MCI/DEM**	**CU**	**MCI/DEM**
Cognitive Measures										
Remote	rho	rho	rho	rho	rho	rho	rho	rho	rho	rho
MTD-SBCr	−0.24***	−0.52***	−0.21***	−0.35*	−0.23***	−0.40**	0.18***	0.54***	−0.32***	−0.15
MTD-SBCz	−0.23***	−0.53***	−0.21***	−0.38*	−0.23***	−0.42**	0.17***	0.52***	−0.31***	−0.15
SLS Sum of Trials	−0.19***	−0.38*	−0.19***	−0.28	−0.22***	−0.35*	0.14***	0.55***	−0.26***	−0.23
SYM	0.25***	0.49**	0.12**	0.30	0.12**	0.26	0.21***	−0.24	0.36***	0.17
SYMaw	−0.27***	−0.51***	−0.13***	−0.33*	−0.13**	−0.29	0.21***	0.27	−0.37***	−0.17
In-Person										
Mayo-PACC	−0.28***	−0.36*	−0.11**	−0.05	−0.12**	−0.05	0.22***	0.26	−0.41***	−0.30
Global Cognition Z	−0.28***	−0.40*	−0.12**	−0.03	−0.14***	−0.00	0.27***	0.21	−0.40***	−0.32
AVLT Sum of Trials	−0.19***	−0.54***	−0.09*	−0.41*	−0.12**	−0.39*	0.15***	0.47**	−0.27***	−0.21
Trail Making Test B	−0.29***	0.33*	0.14***	−0.01	0.13**	0.00	0.22***	−0.13	−0.44***	0.15
Digit Symbol Coding	−0.28***	−0.30	−0.14***	0.14	−0.12**	0.25	0.22***	0.11	−0.39***	−0.22
STMS	−0.15***	0.12	−0.09*	−0.07	−0.10*	−0.09	0.17***	0.04	−0.26***	0.04

*p<.05; **p<.01; ***p<.001; Note: AVLT = Auditory Verbal Learning Test; AVLT Sum of Trials = AVLT 1–5 total + Trial 6 + 30-minute delay; CU = Cognitively Unimpaired; Digit Symbol Coding = WAIS-R Digit Symbol Substitution Test; DEM =Dementia; EC = entorhinal cortex; Global Cognition z = average z across all neuropsychological tests administered during an in-person visit; Mayo-PACC = Mayo Preclinical Alzheimer’s disease Cognitive Composite (average z of Auditory Verbal Learning Test sum of trials, animal fluency, and inversed Trails B); MCI = Mild Cognitive Impairment; MTD = Mayo Test Drive; MTD-SBCr = Mayo Test Drive Screening Battery Composite raw (Stricker Learning Span sum of trials + Symbols Test accuracy-weighted average correct items response time); MTD-SBCz = Mayo Test Drive Screening Battery Composite z; PET = Positron Emission Tomography; ROI = region of interest; SLS = Stricker Learning Span; SLS Sum of Trials = SLS 1–5 total + delay; STMS = Short Test of Mental Status, which is similar to the MMSE, is included for reference; SUVR = Standard Uptake Volume Ratio; SYM = Symbols Test average correct item response time; SYMaw= Symbols Test accuracy-weighted average correct item response time; Trail Making Test B = Trail Making Test B completion time; WMH = white matter hyperintensities.

## Discussion

In this study of 684 older adults who were predominantly CU, we showed that worse performances on MTD, as measured via a raw composite score (MTD-SBCr) derived from the SLS and Symbols tests, was significantly associated with higher levels of amyloid in a PET meta-ROI, higher levels of tau in the entorhinal cortex specifically as well as in a PET meta-ROI, lower HV, and higher WMH. There were also significant associations across all imaging biomarkers with MTD-SBCz, as well as the SLS Sum of Trials, SYM, and SYMaw metrics. For reference, Mayo-PACC and Global Cognition z, two person-administered cognitive composites, were also associated with all imaging biomarkers with the exception of the tau PET meta-ROI. The AVLT (similar to the SLS) and Trail Making Test B (similar to the Symbols test) were both significantly associated with all imaging biomarkers, whereas Digit Symbol Coding (also similar to the Symbols test) was only associated with HV and WMH. Additionally, we demonstrated that MTD performances for both composites (MTD-SBCr and MTD-SBCz) differ by diagnostic category (CU, MCI, dementia) in an expected fashion, and that associations between MTD and imaging biomarkers are generally stronger for those diagnosed with MCI or dementia compared with CU individuals, again as expected. MTD and in-person neuropsychological measures were robustly associated with each other, as well. Together, these findings highlight MTD’s ability to detect cognitive change in a large group of predominantly (94%) CU individuals that is associated with greater levels of amyloid and tau burden, consistent with the biological diagnosis of AD, as well as hippocampal and WMH volumes, together which supports MTD’s criterion validity. Given that the MTD-SBCr additionally demonstrated the ability to differentiate diagnostic groups, it has promising utility as a generalizable tool for detecting subtle objective cognitive impairment that is independent of study-specific samples. Overall, findings from this study highlight the ability of MTD to detect subtle cognitive change associated with biomarkers of AD and neurodegeneration across diagnostic groups in a remote, self-administered digital fashion.

Remote, digital cognitive assessments have been developing rapidly over the past few years as older adults have become more accustomed to digital technology, as its effectiveness has been shown in reaching large groups of people who may otherwise have limited access to cognitive assessment, and due to its necessity during the COVID-19 pandemic (34). To date, most of these remote digital assessments utilize multiple assessments, either once per day over several days (e.g., BRANCH; although this can be used at a single time point) (54), or multiple times per day over several days (e.g., Mobile Monitoring of Cognitive Change; M2C2) (55). Performance on BRANCH has robust associations with the PACC5 as well as associations with cortical amyloid and entorhinal tau deposition (12), with diminished seven-day practice effects on BRANCH linked to increased levels of amyloid and declines on the PACC5 over one year in CU individuals (54). Similarly, diminished practice effects on C3 over a three month period have been associated with greater amyloid and tau burden as well as steeper annual decline on the PACC5 (56). Another remote digital assessment – the M2C2 Prices task as part of the NIH Mobile Toolbox – distinguishes A+ versus A- participants better than the MoCA (55). Our group has previously shown that a one-time administered SLS discriminates between A+ and A− groups, as well as A+T+ and A−T− groups in a predominantly CU sample (16). In this study, we further demonstrated that worse performance on the MTD-SBCr and MTD-SBCz were both continuously associated with higher amyloid, tau, and WMH burden, and lower HV. By including these additional biomarkers in this study of predominantly CU individuals, we were able to demonstrate the sensitivity of MTD in detecting subtle cognitive change was not only associated with amyloid, but with other AD-related biomarkers, as well. MTD uses a one-time administration, rather than repeated measurements as some other remote digital assessments require currently, and is able to detect these biomarker-associated cognitive changes in CU individuals. Additionally, MTD’s associations with these imaging biomarkers are broadly comparable to the continuous associations between the in person-administered Mayo-PACC and imaging biomarkers.

The components that comprise the two MTD composites – the SLS and Symbols Test – were both individually associated with all imaging biomarkers investigated in this study. These associations were similar to their person-administered cognitive test counterparts that were provided for reference in this study – the AVLT, Trail Making Test B, and Digit Symbol Coding, with the AVLT and Trail Making Test B also associated with all imaging biomarkers, and Digit Symbol Coding associated with HV and WMH. We did not directly compare associations between MTD and imaging biomarkers with traditional person-administered cognitive measures and imaging biomarkers, as the SLS has already been directly compared to the AVLT in its ability to predict dichotomous AD biomarker status (16). Our goal was to investigate continuous associations, for which we prioritized inclusion of all participants with imaging data as opposed to limiting the sample to those with all cognitive measures which would have excluded most individuals with dementia due to test battery differences across the MCSA and ADRC. In particular, most dementia participants are missing data for Global Cognition z given differences in MCSA and ADRC test batteries.

One challenge with PACCs is that findings are typically study-specific, with results typically depicted as z-scores derived from the sample itself. We developed the MTD-SBCr to move beyond study-specific scores and cutoffs to develop a score that can be interpreted independent of study sample. We demonstrated that the MTD-SBCr, which does not rely on study-specific z-scores, was able to distinguish between CU, MCI, and dementia diagnostic groups with large effect sizes. Additionally, when separated by diagnostic groups, MTD-SBCr was significantly correlated with all imaging biomarkers, with stronger associations for MCI and dementia diagnostic categories. As such, the MTD-SBCr is sensitive to diagnostic categories associated with AD-related biomarkers. Given that it does not rely on study-specific performances like z-scores for interpretation, a future direction of MTD-SBCr is to develop clinical cut-offs of performance using Area Under the Receiving Operative Curve analyses with associated sensitivity and specificity data such that raw scores can have meaning associated with diagnostic categories. The MTD-SBCr could also be useful because it does not adjust for age-related effects. We now know that age-related cognitive change may actually be due to previously undetected neuropathological change (21, 57), so adjusting for age-related associations may subtract meaning from cognitive scores. As such, the MTD-SBCr would allow for understanding of cognitive status in clinical settings independent of these factors, and in the future following development of clinical cut-offs, could allow for early screening, triage, and routing for specialty care in a similar fashion to the MMSE or MoCA.

There are several limitations to this study. First, the MCSA is a population-based study that represents the demographic makeup of Rochester, MN, which is primarily comprised of non-Hispanic white individuals. As such, inclusion of individuals from underrepresented racial and ethnic groups is a clear weakness. To address this limitation, we are actively expanding the use of MTD in underrepresented groups including the Jacksonville, FL location of the Mayo ADRC and the University of Mississippi Medical Center in Jackson, MS, to promote targeted recruitment of participants from underrepresented groups to our study sample, with a particular focus on Black participants given this population’s increased risk for dementia. We are also nearing completion of a Spanish language adaptation of MTD following a community-engaged approach that incorporated focus group feedback from native Spanish speakers (58), which will expand our ability to include Spanish-speaking individuals in future work given the U.S. Hispanic population’s increased risk for dementia, as well. Second, while the focus of this study was detecting biomarker-cognition associations in the earlier clinical stages of the AD continuum, inclusion of more participants with MCI and/or dementia could have allowed for even stronger associations between AD-related biomarkers and MTD performance and may have allowed for better characterization of MTD performance in these diagnostic groups. Third, both the MTD-SBCz and MTD-SBCr are weighted toward the SLS due to how they are calculated. While this approach aligns with research suggesting memory is one of the earliest changes observed in the typical Alzheimer’s disease process (59), future work should examine whether alternative data-driven approaches to creating a composite could outperform this theoretical a priori approach, and adaptations may be needed in atypical AD populations or for other neurodegenerative or medical conditions. We previously demonstrated that a 3-measure person-administered composite (Mayo-PACC) performed as well as a Global Cognition z-score (9 tests from 4 cognitive domains) in terms of sensitivity to longitudinal amyloid-related cognitive decline (10); future work is needed to similarly compare the MTD composites, which are comprised only of measures of memory and processing speed/executive function, to composites that are inclusive of more cognitive domains. The adaptive nature of the SLS may raise concerns that individuals can receive a different version of the test based on variations in within-test performance. However, other computer adaptive memory tests including the National Institutes of Health Toolbox Picture Sequencing Test and Cambridge Neuropsychological Test Automated Battery (CANTAB) Paired Associate Learning Task share similar concerns, and our prior finding that the computer-adaptive SLS was comparable to the person-administered AVLT in differentiating AD-biomarker groups helps support that criterion validity is maintained with an adaptive testing approach (15). Fourth, the time gap between collection of MTD and neuroimaging data was 5.75 months on average; while it is possible that biomarker levels may have changed in that interim time frame potentially impacting our findings, markers of amyloid and tau typically show relative stability within this timeframe (60). Fifth, it is possible that selection bias may impact findings, as those who chose to participate in the ancillary MTD study may have been more comfortable with technology than those who did not participate. We worked to help reduce this bias by allowing MTD to be completed in clinic upon request to help ensure those without necessary technology or limited technology proficiency could participate, but few participants in the current sample took advantage of this in-clinic option (n=3). Finally, it is also important to note that the remote nature of MTD allows for variability in the testing environment, possible interference, unknown trouble navigating the technology, other technical difficulties, or the possibility of unanticipated assistance during the assessment. Future work is needed to examine the frequency and potential impact of these factors on results. Similarly, the utility of any remote assessment must be evaluated in the context of the platform’s usability. We previously presented preliminary data within the MCSA and ADRC that supported high completion rates of MTD once a session was initiated that were similar for individuals with and without cognitive impairment (16). Despite comparable MTD completion rates across those with and without cognitive impairment, participation rates among those with cognitive impairment were lower than for CU individuals in that pilot study sample, similar to patterns of participation and willingness to engage in remote assessment seen in other studies (61, 62). Future work will provide an expanded update of participation rates, usability data, and feasibility data for MTD in participants with and without available neuroimaging data.

This study has notable strengths. We were able to examine associations between multiple imaging biomarkers of Alzheimer’s disease and performance on a remote, digital cognitive assessment composite measure in a relatively large sample. Further, our ability to demonstrate continuous associations between these biomarkers and cognitive performance in a predominantly CU sample suggests promising ability to detect subtle cognitive changes at the earliest stages of the AD biological cascade. Given that MTD is a onetime, approximately 15-minute remote assessment, this provides utility for access and ease for inclusion in clinical trials as well as in clinical settings more broadly. The development of the MTD-SBCr augments this utility, as it could aid interpretation of MTD performance. Furthermore, the nature of the MCSA, which is population based, provides generalizability of our findings because individuals with other comorbidities are not excluded. Our findings are also responsive to the update to the amyloid-tau-neurodegeneration (ATN) research framework (18), as we demonstrate MTD’s association with core AD biomarkers including amyloid and tau PET, HV – a non-specific neurodegeneration biomarker, and WMH indicating common non-AD vascular co-pathology. To our knowledge, demonstrating associations with all of these biomarkers within a single study has not been completed to date with other remote digital assessments.

In summary, our study demonstrates that the MTD composites, comprised of the SLS and the Symbols Test, are associated with continuous measures of imaging biomarkers including amyloid and tau PET, HV, and WMH. These associations are also seen in the individual subtests of MTD – SLS and Symbols Test. Further, the MTD composite differentiates CU, MCI, and dementia diagnostic groups, and associations between MTD and imaging biomarkers are strengthened in MCI and dementia diagnostic groups. Together, our findings highlight the criterion validity of MTD as a remote, digital cognitive assessment linked with AD-related biological change that can be detected even in individuals who are not demonstrating any overt cognitive impairment.

## Supplemental Online Resources

Continuous Associations Between Remote Self-Administered Cognitive Measures and Imaging Biomarkers of Alzheimer’s Disease

## Notes

This manuscript was originally submitted as a pre-print to medRxiv with the following citation: Boots EA, Frank RD, Fan WZ, et al. 2024. Continuous Associations Between Remote Self-Administered Cognitive Measures and Imaging Biomarkers of Alzheimer’s Disease. medRxiv. doi:10.1101/2024.03.01.2430361. A poster presentation of early/partial data from this work in a smaller sample size was presented at the Alzheimer’s Association International Conference (July 2023). Another portion of this work was submitted as an abstract for the American Psychological Association Annual Convention (August 2024). Copyright 2024 Mayo Foundation for Medical Education and Research.

## References

[1] Cummings J, Osse AML, Cammann D, Powell J, Chen J. Anti-Amyloid Monoclonal Antibodies for the Treatment of Alzheimer’s Disease. BioDrugs. Nov 13 2023;doi:10.1007/s40259-023-00633-2 10.1007/s40259-023-00633-2 PMC10789674 37955845

[2] Cummings J, Zhou Y, Lee G, Zhong K, Fonseca J, Cheng F. Alzheimer’s disease drug development pipeline: 2023. Alzheimers Dement (N Y). Apr–Jun 2023;9(2):e12385. doi:10.1002/trc2.12385 37251912 10.1002/trc2.12385 PMC10210334

[3] Livingston G, Huntley J, Sommerlad A, et al. Dementia prevention, intervention, and care: 2020 report of the Lancet Commission. Lancet. Aug 8 2020;396(10248):413–446. doi:10.1016/S0140-6736(20)30367-6 32738937 10.1016/S0140-6736(20)30367-6 PMC7392084

[4] Sabbagh MN, Boada M, Borson S, et al. Rationale for Early Diagnosis of Mild Cognitive Impairment (MCI) Supported by Emerging Digital Technologies. J Prev Alzheimers Dis. 2020;7(3):158–164. doi:10.14283/jpad.2020.19 32463068 10.14283/jpad.2020.19

[5] Sabbagh MN, Boada M, Borson S, et al. Early Detection of Mild Cognitive Impairment (MCI) in an At-Home Setting. J Prev Alzheimers Dis. 2020;7(3):171–178. doi:10.14283/jpad.2020.22 32463070 10.14283/jpad.2020.22

[6] Alawode DOT, Heslegrave AJ, Ashton NJ, et al. Transitioning from cerebrospinal fluid to blood tests to facilitate diagnosis and disease monitoring in Alzheimer’s disease. J Intern Med. Sep 2021;290(3):583–601. doi:10.1111/joim.13332 34021943 10.1111/joim.13332 PMC8416781

[7] Schneider LS, Goldberg TE. Composite cognitive and functional measures for early stage Alzheimer’s disease trials. Alzheimers Dement (Amst). 2020;12(1):e12017. doi:10.1002/dad2.12017 32432155 10.1002/dad2.12017 PMC7233425

[8] Donohue MC, Sperling RA, Salmon DP, et al. The preclinical Alzheimer cognitive composite: measuring amyloid-related decline. JAMA Neurol. Aug 2014;71(8):961–70. doi:10.1001/jamaneurol.2014.803 24886908 10.1001/jamaneurol.2014.803 PMC4439182

[9] Papp KV, Rentz DM, Orlovsky I, Sperling RA, Mormino EC. Optimizing the preclinical Alzheimer’s cognitive composite with semantic processing: The PACC5. Alzheimers Dement (N Y). Nov 2017;3(4):668–677. doi:10.1016/j.trci.2017.10.004 29264389 10.1016/j.trci.2017.10.004 PMC5726754

[10] Stricker NH, Twohy EL, Albertson SM, et al. Mayo-PACC: A parsimonious preclinical Alzheimer’s disease cognitive composite comprised of public-domain measures to facilitate clinical translation. Alzheimers Dement. Jun 2023;19(6):2575–2584. doi:10.1002/alz.12895 36565459 10.1002/alz.12895 PMC10272034

[11] Papp KV, Rentz DM, Maruff P, et al. The Computerized Cognitive Composite (C3) in an Alzheimer’s Disease Secondary Prevention Trial. J Prev Alzheimers Dis. 2021;8(1):59–67. doi:10.14283/jpad.2020.38 33336226 10.14283/jpad.2020.38 PMC7755110

[12] Papp KV, Samaroo A, Chou HC, et al. Unsupervised mobile cognitive testing for use in preclinical Alzheimer’s disease. Alzheimers Dement (Amst). 2021;13(1):e12243. doi:10.1002/dad2.12243 34621977 10.1002/dad2.12243 PMC8481881

[13] Farrell ME, Papp KV, Buckley RF, et al. Association of Emerging beta-Amyloid and Tau Pathology With Early Cognitive Changes in Clinically Normal Older Adults. Neurology. Apr 12 2022;98(15):e1512–e1524. doi:10.1212/WNL.0000000000200137 35338074 10.1212/WNL.0000000000200137 PMC9012271

[14] Stricker NH, Stricker JL, Karstens AJ, et al. A novel computer adaptive word list memory test optimized for remote assessment: Psychometric properties and associations with neurodegenerative biomarkers in older women without dementia. Alzheimers Dement (Amst). 2022;14(1):e12299. doi:10.1002/dad2.12299 35280963 10.1002/dad2.12299 PMC8905660

[15] Stricker JL, Corriveau-Lecavalier N, Wiepert DA, Botha H, Jones DT, Stricker NH. Neural network process simulations support a distributed memory system and aid design of a novel computer adaptive digital memory test for preclinical and prodromal Alzheimer’s disease. Neuropsychology. Sep 2023;37(6):698–715. doi:10.1037/neu0000847 36037486 10.1037/neu0000847 PMC9971333

[16] Stricker NH, Stricker JL, Frank RD, et al. Stricker Learning Span criterion validity: a remote self-administered multi-device compatible digital word list memory measure shows similar ability to differentiate amyloid and tau PET-defined biomarker groups as in-person Auditory Verbal Learning Test. J Int Neuropsychol Soc. Jun 30 2023:1–14. doi:10.1017/S1355617723000322 10.1017/S1355617723000322 PMC10756923 37385974

[17] Patel JS, Christianson TJ, Karstens AJ, et al. An examination of the usability of the Mayo Test Drive remote cognitive testing platform in older adults with and without cognitive impairment. Alzheimer’s & Dementia. 2022;18(S7):e061834. doi:10.1002/alz.061834

[18] Jack CR, Jr., Bennett DA, Blennow K, et al. NIA-AA Research Framework: Toward a biological definition of Alzheimer’s disease. Alzheimers Dement. Apr 2018;14(4):535–562. doi:10.1016/j.jalz.2018.02.018 29653606 10.1016/j.jalz.2018.02.018 PMC5958625

[19] Insel PS, Donohue MC, Sperling R, Hansson O, Mattsson-Carlgren N. The A4 study: beta-amyloid and cognition in 4432 cognitively unimpaired adults. Ann Clin Transl Neurol. May 2020;7(5):776–785. doi:10.1002/acn3.51048 32315118 10.1002/acn3.51048 PMC7261742

[20] Papp KV, Rofael H, Veroff AE, et al. Sensitivity of the Preclinical Alzheimer’s Cognitive Composite (PACC), PACC5, and Repeatable Battery for Neuropsychological Status (RBANS) to Amyloid Status in Preclinical Alzheimer’s Disease -Atabecestat Phase 2b/3 EARLY Clinical Trial. J Prev Alzheimers Dis. 2022;9(2):255–261. doi:10.14283/jpad.2022.17 35542998 10.14283/jpad.2022.17

[21] Boyle PA, Wang T, Yu L, et al. To what degree is late life cognitive decline driven by age-related neuropathologies? Brain. Aug 17 2021;144(7):2166–2175. doi:10.1093/brain/awab092 33742668 10.1093/brain/awab092 PMC8370442

[22] Jack CR, Jr., Therneau TM, Weigand SD, et al. Prevalence of Biologically vs Clinically Defined Alzheimer Spectrum Entities Using the National Institute on Aging-Alzheimer’s Association Research Framework. JAMA Neurol. Jul 15 2019;76(10):1174–83. doi:10.1001/jamaneurol.2019.1971 31305929 10.1001/jamaneurol.2019.1971 PMC6632154

[23] Alden EC, Pudumjee SB, Lundt ES, et al. Diagnostic accuracy of the Cogstate Brief Battery for prevalent MCI and prodromal AD (MCI A(+) T(+)) in a population-based sample. Alzheimers Dement. Apr 2021;17(4):584–594. doi:10.1002/alz.12219 33650308 10.1002/alz.12219 PMC8371696

[24] Alden EC, Lundt ES, Twohy EL, et al. Mayo normative studies: A conditional normative model for longitudinal change on the Auditory Verbal Learning Test and preliminary validation in preclinical Alzheimer’s disease. Alzheimers Dement (Amst). 2022;14(1):e12325. doi:10.1002/dad2.12325 35860792 10.1002/dad2.12325 PMC9286327

[25] Stricker NH, Christianson TJ, Lundt ES, et al. Mayo Normative Studies: Regression-Based Normative Data for the Auditory Verbal Learning Test for Ages 30–91 Years and the Importance of Adjusting for Sex. J Int Neuropsychol Soc. Mar 2021;27(3):211–226. doi:10.1017/S1355617720000752 32815494 10.1017/S1355617720000752 PMC7895855

[26] St Sauver JL, Grossardt BR, Yawn BP, et al. Data resource profile: the Rochester Epidemiology Project (REP) medical records-linkage system. Int J Epidemiol. Dec 2012;41(6):1614–24. doi:10.1093/ije/dys195 23159830 10.1093/ije/dys195 PMC3535751

[27] Morris JC. The Clinical Dementia Rating (CDR): Current version and scoring rules. Neurology. Nov 1993;43(11):2412–2414. doi:10.1212/WNL.43.11.2412-a 8232972 10.1212/wnl.43.11.2412-a

[28] Roberts RO, Geda YE, Knopman DS, et al. The Mayo Clinic Study of Aging: design and sampling, participation, baseline measures and sample characteristics. Neuroepidemiology. 2008;30(1):58–69. doi:10.1159/000115751 18259084 10.1159/000115751 PMC2821441

[29] Petersen RC. Mild cognitive impairment as a diagnostic entity. J Intern Med. Sep 2004;256(3):183–94. doi:10.1111/j.1365-2796.2004.01388.x 15324362 10.1111/j.1365-2796.2004.01388.x

[30] American Psychiatric Association. Diagnostic and Statistical Manual of Mental Disorders (DSM-IV). 4th ed. American Psychiatric Association; 1994.

[31] Sliwinski MJ, Mogle JA, Hyun J, Munoz E, Smyth JM, Lipton RB. Reliability and Validity of Ambulatory Cognitive Assessments. Assessment. Jan 2018;25(1):14–30. doi:10.1177/1073191116643164 27084835 10.1177/1073191116643164 PMC5690878

[32] Nicosia J, Aschenbrenner AJ, Balota DA, et al. Unsupervised high-frequency smartphone-based cognitive assessments are reliable, valid, and feasible in older adults at risk for Alzheimer’s disease. J Int Neuropsychol Soc. Jun 2023;29(5):459–471. doi:10.1017/S135561772200042X 36062528 10.1017/S135561772200042X PMC9985662

[33] Weintraub S, Carrillo MC, Farias ST, et al. Measuring cognition and function in the preclinical stage of Alzheimer’s disease. Alzheimers Dement (N Y). 2018;4:64–75. doi:10.1016/j.trci.2018.01.003 29955653 10.1016/j.trci.2018.01.003 PMC6021264

[34] Ohman F, Hassenstab J, Berron D, Scholl M, Papp KV. Current advances in digital cognitive assessment for preclinical Alzheimer’s disease. Alzheimers Dement (Amst). 2021;13(1):e12217. doi:10.1002/dad2.12217 34295959 10.1002/dad2.12217 PMC8290833

[35] Stricker NH, Twohy EL, Albertson SM, et al. Diagnostic accuracy of the Stricker Learning Span and Mayo Test Drive Composite for amnestic Mild Cognitive Impairment. Alzheimer’s & Dementia. 2022;18(S7):e063190. doi:10.1002/alz.063190

[36] Ivnik RJ, Malec JF, Smith GE, et al. Mayo’s older americans normative studies: Updated AVLT norms for ages 56 to 97. Clinical Neuropsychologist. 1992/06/01 1992;6(sup001):83–104. doi:10.1080/13854049208401880

[37] Wechsler D. Wechsler Memory Scale-Revised. Psychological Corporation; 1987.

[38] Kaplan EF, Goodglass H, Weintraub S. The Boston Naming Test. 2nd ed. Lea & Febiger; 1982.

[39] Lucas JA, Ivnik RJ, Smith GE, et al. Mayo’s older Americans normative studies: category fluency norms. J Clin Exp Neuropsychol. Apr 1998;20(2):194–200. doi:10.1076/jcen.20.2.194.1173 9777473 10.1076/jcen.20.2.194.1173

[40] Reitan RM. Validity of the Trail Making Test as an Indicator of Organic Brain Damage. Perceptual and Motor Skills. 1958;8(3):271–276. doi:10.2466/pms.1958.8.3.271

[41] Wechsler D. Wechsler Adult Intellience Scale-Revised. Psychological Corporation; 1987.

[42] Kokmen E, Naessens JM, Offord KP. A short test of mental status: description and preliminary results. Mayo Clin Proc. Apr 1987;62(4):281–8. doi:10.1016/s0025-6196(12)61905-3 3561043 10.1016/s0025-6196(12)61905-3

[43] Tang-Wai DF, Knopman DS, Geda YE, et al. Comparison of the short test of mental status and the mini-mental state examination in mild cognitive impairment. Arch Neurol. Dec 2003;60(12):1777–81. doi:10.1001/archneur.60.12.1777 14676056 10.1001/archneur.60.12.1777

[44] Townley RA, Syrjanen JA, Botha H, et al. Comparison of the Short Test of Mental Status and the Montreal Cognitive Assessment Across the Cognitive Spectrum. Mayo Clin Proc. Aug 2019;94(8):1516–1523. doi:10.1016/j.mayocp.2019.01.043 31280871 10.1016/j.mayocp.2019.01.043 PMC6937135

[45] Jack CR, Jr., Lowe VJ, Senjem ML, et al. 11C PiB and structural MRI provide complementary information in imaging of Alzheimer’s disease and amnestic mild cognitive impairment. Brain. Mar 2008;131 (Pt 3):665–80. doi:10.1093/brain/awm336 18263627 10.1093/brain/awm336 PMC2730157

[46] Jack CR, Jr., Wiste HJ, Weigand SD, et al. Defining imaging biomarker cut points for brain aging and Alzheimer’s disease. Alzheimers Dement. Mar 2017;13(3):205–216. doi:10.1016/j.jalz.2016.08.005 27697430 10.1016/j.jalz.2016.08.005 PMC5344738

[47] Vemuri P, Lowe VJ, Knopman DS, et al. Tau-PET uptake: Regional variation in average SUVR and impact of amyloid deposition. Alzheimers Dement (Amst). 2017;6:21–30. doi:10.1016/j.dadm.2016.12.010 28138510 10.1016/j.dadm.2016.12.010 PMC5257031

[48] Lowe VJ, Bruinsma TJ, Wiste HJ, et al. Cross-sectional associations of tau-PET signal with cognition in cognitively unimpaired adults. Neurology. Jul 2 2019;93(1):e29–e39. doi:10.1212/WNL.0000000000007728 31147421 10.1212/WNL.0000000000007728 PMC6659005

[49] Ashburner J, Friston KJ. Unified segmentation. Neuroimage. Jul 1 2005;26(3):839–51. doi:10.1016/j.neuroimage.2005.02.018 15955494 10.1016/j.neuroimage.2005.02.018

[50] Avants BB, Tustison NJ, Song G, Cook PA, Klein A, Gee JC. A reproducible evaluation of ANTs similarity metric performance in brain image registration. Neuroimage. Feb 1 2011;54(3):2033–44. doi:10.1016/j.neuroimage.2010.09.025 20851191 10.1016/j.neuroimage.2010.09.025 PMC3065962

[51] Schwarz CG, Gunter JL, Wiste HJ, et al. A large-scale comparison of cortical thickness and volume methods for measuring Alzheimer’s disease severity. Neuroimage Clin. 2016;11:802–812. doi:10.1016/j.nicl.2016.05.017 28050342 10.1016/j.nicl.2016.05.017 PMC5187496

[52] Cogswell PM, Aakre JA, Castillo AM, et al. Population-Based Prevalence of Infarctions on 3D Fluid-Attenuated Inversion Recovery (FLAIR) Imaging. J Stroke Cerebrovasc Dis. Aug 2022;31(8):106583. doi:10.1016/j.jstrokecerebrovasdis.2022.106583 35689933 10.1016/j.jstrokecerebrovasdis.2022.106583 PMC9329259

[53] Graff-Radford J, Arenaza-Urquijo EM, Knopman DS, et al. White matter hyperintensities: relationship to amyloid and tau burden. Brain. Aug 1 2019;142(8):2483–2491. doi:10.1093/brain/awz162 31199475 10.1093/brain/awz162 PMC6658846

[54] Papp KV, Jutten RJ, Soberanes D, et al. Early detection of amyloid-related changes in memory among cognitively unimpaired older adults with daily digital testing. Ann Neurol. Nov 22 2023;doi:10.1002/ana.26833 10.1002/ana.26833 PMC10922126 37991080

[55] Thompson LI, Kunicki ZJ, Emrani S, et al. Remote and in-clinic digital cognitive screening tools outperform the MoCA to distinguish cerebral amyloid status among cognitively healthy older adults. Alzheimers Dement (Amst). Oct–Dec 2023;15(4):e12500. doi:10.1002/dad2.12500 38026761 10.1002/dad2.12500 PMC10680059

[56] Jutten RJ, Rentz DM, Fu JF, et al. Monthly At-Home Computerized Cognitive Testing to Detect Diminished Practice Effects in Preclinical Alzheimer’s Disease. Front Aging Neurosci. 2021;13:800126. doi:10.3389/fnagi.2021.800126 35095476 10.3389/fnagi.2021.800126 PMC8792465

[57] Bos I, Vos SJB, Jansen WJ, et al. Amyloid-beta, Tau, and Cognition in Cognitively Normal Older Individuals: Examining the Necessity to Adjust for Biomarker Status in Normative Data. Front Aging Neurosci. 2018;10:193. doi:10.3389/fnagi.2018.00193 29988624 10.3389/fnagi.2018.00193 PMC6027060

[58] Karstens AJ, Aduen P, Luna NA, et al. Adaptation of Mayo Test Drive for Monolingual and Bilingual Spanish Speakers Using a Community Engaged Approach. presented at: International Neuropsychological Society 52nd Annual Meeting; 2024; New York, NY.

[59] Caselli RJ, Locke DE, Dueck AC, et al. The neuropsychology of normal aging and preclinical Alzheimer’s disease. Alzheimers Dement. Jan 2014;10(1):84–92. doi:10.1016/j.jalz.2013.01.004 23541188 10.1016/j.jalz.2013.01.004 PMC3700591

[60] Jack CR, Jr., Wiste HJ, Therneau TM, et al. Associations of Amyloid, Tau, and Neurodegeneration Biomarker Profiles With Rates of Memory Decline Among Individuals Without Dementia. JAMA. Jun 18 2019;321(23):2316–2325. doi:10.1001/jama.2019.7437 31211344 10.1001/jama.2019.7437 PMC6582267

[61] Jacobs DM, Peavy GM, Banks SJ, Gigliotti C, Little EA, Salmon DP. A survey of smartphone and interactive video technology use by participants in Alzheimer’s disease research: Implications for remote cognitive assessment. Alzheimers Dement (Amst). 2021;13(1):e12188. doi:10.1002/dad2.12188 34027018 10.1002/dad2.12188 PMC8132053

[62] Ashford MT, Aaronson A, Kwang W, et al. Unsupervised Online Paired Associates Learning Task from the Cambridge Neuropsychological Test Automated Battery (CANTAB(R)) in the Brain Health Registry. J Prev Alzheimers Dis. 2024;11(2):514–524. doi:10.14283/jpad.2023.117 38374758 10.14283/jpad.2023.117 PMC10879687

