# Supplementary material for: Continuous Associations between Remote Self-Administered Cognitive Measures and Imaging Biomarkers of Alzheimer’s Disease
Source: J Prev Alzheimers Dis. 2024 May 29;11(5):1467–79. doi: 10.14283/jpad.2024.99 (PMC11436415; doi:10.14283/jpad.2024.99)
Supplement: Supplementary file 1 — Continuous Associations Between Remote Self-Administered Cognitive Measures and Imaging Biomarkers of Alzheimer’s Disease [file 42414_2024_99_MOESM1_ESM.docx]

**Continuous Associations Between Remote Self-Administered Cognitive Measures and Imaging Biomarkers of Alzheimer’s Disease**

Supplemental Online Resources

*Journal of Prevention of Alzheimer’s Disease*

Elizabeth A. Boots, Ph.D.,^1^ Ryan D. Frank, M.S.,^2^ Winnie Z. Fan, M.S., ^2^ Teresa J. Christianson, M.S., ^2^ Walter K. Kremers, Ph.D., ^2^ John L. Stricker, Ph.D., ^3^ Mary M. Machulda, Ph.D., ^1^ Julie A. Fields, Ph.D., ^1^ Jason Hassenstab, PhD., ^4^ Jonathan Graff-Radford, M.D., ^5^ Prashanthi Vemuri, Ph.D., ^6^ Clifford R. Jack, M.D.,^6^ David S. Knopman, M.D., ^5^ Ronald C. Petersen, M.D., Ph.D., ^5^ Nikki H. Stricker, Ph.D.^1^

^1^Divison of Neurocognitive Disorders, Department of Psychiatry and Psychology, Mayo Clinic, Rochester, MN, USA

^2^Division of Biomedical Statistics and Informatics, Department of Quantitative Health Sciences, Mayo Clinic, Rochester, Minnesota, USA

^3^ Department of Information Technology, Mayo Clinic, Rochester, Minnesota, USA

^4^Department of Neurology and Psychological & Brain Sciences, Washington University in St. Louis, St. Louis, Missouri, USA

^5^ Department of Neurology, Mayo Clinic, Rochester, Minnesota, USA

^6^ Department of Radiology, Mayo Clinic, Rochester, Minnesota, USA

Corresponding Author: Nikki H. Stricker, Ph.D., ABPP-CN, Mayo Clinic, 200 First Street SW, Rochester, MN 55905; 507-284-2649 (phone), 507-284-4158 (fax), [stricker.nikki@mayo.edu](mailto:stricker.nikki@mayo.edu) (email).

Copyright 2024 Mayo Foundation for Medical Education and Research, all rights reserved.

*Supplementary Table 1*. Spearman Correlations between remote and in-person cognitive measures.

|  | **Remote Mayo Test Drive Measures** | | | | | | | | | |
| --- | --- | --- | --- | --- | --- | --- | --- | --- | --- | --- |
|  | MTD-SBCr | | MTD-SBCz | | SLS Sum of Trials | | SYM | | SYM_aw_ | |
| **In-Person Cognitive Measures** | rho | N | rho | N | rho | N | rho | N | rho | N |
| Mayo-PACC | 0.68 *** | 657 | 0.68 *** | 657 | 0.60 *** | 658 | -0.60 *** | 658 | 0.59 *** | 658 |
| Global Cognition Z | 0.67 *** | 626 | 0.67 *** | 626 | 0.59 *** | 627 | -0.59 *** | 627 | 0.58 *** | 627 |
| AVLT Sum of Trials | 0.64 *** | 668 | 0.65 *** | 668 | 0.61 *** | 669 | -0.42 *** | 669 | 0.43 *** | 669 |
| Trail Making Test B | -0.57 *** | 665 | -0.57 *** | 664 | -0.46 *** | 666 | 0.62 *** | 666 | -0.61 *** | 666 |
| Digit Symbol Coding | 0.55 *** | 654 | 0.56 *** | 654 | 0.44 *** | 655 | -0.63 *** | 655 | 0.59 *** | 655 |
| STMS | 0.56 *** | 673 | 0.55 *** | 673 | 0.49 *** | 674 | -0.49 *** | 674 | 0.50 *** | 674 |

*p<.05; **p<.01; ***p<.001

Note: AVLT = Auditory Verbal Learning Test; AVLT Sum of Trials = AVLT 1-5 total + Trial 6 + 30-minute delay; Digit Symbol Coding = WAIS-R Digit Symbol Substitution Test; Global Cognition z = average z across all neuropsychological tests administered during an in-person visit; Mayo-PACC = Mayo Preclinical Alzheimer’s disease Cognitive Composite (average z of Auditory Verbal Learning Test sum of trials, animal fluency, and inversed Trails B); MTD-SBCr = Mayo Test Drive Screening Battery Composite raw (Stricker Learning Span sum of trials + Symbols Test accuracy-weighted average correct items response time); MTD-SBCz = Mayo Test Drive Screening Battery Composite z; SLS = Stricker Learning Span; SLS Sum of Trials = SLS 1-5 total + delay; STMS = Short Test of Mental Status, which is similar to the MMSE, is included for reference; SYM = Symbols Test average correct item response time; SYM_aw_= Symbols Test accuracy-weighted average correct item response time; Trail Making Test B = Trail Making Test B completion time.

*Supplemental Table 2.* Participant Characteristics with Comparisons between Cognitively Unimpaired, Mild Cognitive Impairment, and Dementia Participants

| **Characteristic*** | **CU** | **MCI** | **CU vs MCI** | | **Dementia** | **CU vs Dementia** | | **MCI vs Dementia** | |
| --- | --- | --- | --- | --- | --- | --- | --- | --- | --- |
|  | N=643 | N=34 | p^†^ | Hedge’s g | N=7 | p^†^ | Hedge’s g | p^†^ | Hedge’s g |
| **Demographics** |  |  |  |  |  |  |  |  |  |
| Age at MCSA/ADRC visit, years | 69.890  (11.111) | 78.809  (10.256) | <0.001 | 0.81  (0.46, 1.15) | 72.820  (8.148) | 0.49 | 0.26  (-0.48, 1.01) | 0.16 | -0.60  (-1.43, 0.22) |
| Sex, N (%) Female | 318  (49.5%) | 19  (55.9%) | 0.47^‡^ | -- | 3  (42.9%) | 1.00^‡^ | -- | 0.68^‡^ | -- |
| Education, years | 15.719  (2.335) | 14.206  (2.649) | <0.001 | -0.64  (-0.99, -0.30) | 16.000  (3.317) | 0.75 | 0.12  (-0.62, 0.86) | 0.13 | 0.65  (-0.18, 1.48) |
| Race, N (%) White | 624  (97.0%) | 33  (97.1%) | 1.00^§^ | -- | 7  (100.0%) | 0.64^§^ | -- | 1.00^§^ | -- |
| Ethnicity, N (%) Non-Hispanic | 639  (99.4%) | 34  (100.0%) | 1.00^§^ | -- | 7  (100.0%) | 1.00^§^ | -- | -- | -- |
| In-person visit to MTD, months | 0.709  (1.896) | 1.308  (3.093) | 0.08 | 0.30  (-0.04, 0.65) | 0.493  (0.404) | 0.76 | -0.11  (-0.86, 0.63) | 0.49 | -0.29  (-1.10, 0.53) |
| MTD to imaging, months | 6.113  (11.622) | -0.447  (2.988) | 0.001 | -0.58  (-0.92, -0.23) | 2.079  (5.102) | 0.36 | -0.35  (-1.09, 0.40) | 0.08 | 0.74  (-0.09, 1.57) |
| MTD completed in clinic, N (%) | 3  (0.5%) | 0  (0.0%) | 1.00^§^ | -- | 0  (0.0%) | 1.00^§^ | -- | -- | -- |
| **Cognition: Mayo Test Drive**^1,2^ |  |  |  |  |  |  |  |  |  |
| MTD-SBCr, n=680 | 107.653  (20.171) | 70.730  (21.322) | <0.001 | -1.83  (-2.19, -1.46) | 45.492  (33.013) | <0.001 | -3.06  (-3.82, -2.29) | 0.01 | -1.07  (-1.92, -0.22) |
| MTD-SBCz, n=680 | 0.000  (1.000) | -1.830  (1.057) | <0.001 | -1.90  (-2.26, -1.53) | -3.082  (1.637) | <0.001 | -3.23  (-4.00, -2.47) | 0.01 | -1.08  (-1.93, -0.23) |
| SLS Sum of Trials, n=681 | 75.833  (17.174) | 48.030 (17.853) | <0.001 | -1.62  (-1.98, -1.26) | 30.857  (20.708) | <0.001 | -2.61  (-3.37, -1.85) | 0.03 | -0.94  (-1.78, -0.09) |
| SYM, n=681 | 3.337  (1.067) | 5.276  (2.207) | <0.001 | 1.69  (1.33, 2.05) | 7.261  (3.358) | <0.001 | 3.53  (2.77, 4.30) | 0.06 | 0.82  (-0.02, 1.65) |
| SYM_aw_, n=681 | 31.839  (6.065) | 22.700  (9.277) | <0.001 | -1.46  (-1.82, -1.10) | 14.635  (13.158) | <0.001 | -2.79  (-3.55, -2.03) | 0.06 | -0.81  (-1.64, 0.03) |
| **Cognition: In-Person Measures**^2,3^ |  |  |  |  |  |  |  |  |  |
| Mayo-PACC z, n=661 | 0.000  (1.000) | -2.356  (1.368) | <.001 | -2.31  (-2.69, -1.93) | -2.071  (1.057) | <.001 | -2.07  (-3.06, -1.08) | 0.49 | 0.21  (-0.83, 1.25) |
| Global Cognition z, n=629 | 0.000  (1.000) | -2.694  (0.923) | <.001 | -2.70  (-3.12, -2.28) | -- | -- | -- | -- | -- |
| AVLT Sum of Trials, n=668 | 66.979  (17.568) | 36.813  (11.255) | <.001 | -1.74  (-2.11, -1.37) | 27.167  (6.113) | 0.001 | -2.27  (-3.09, -1.46) | 0.81 | -0.90  (-1.80, -0.01) |
| Trail Making Test B, n=669 | 72.472  (34.875) | 158.469  (82.729) | <.001 | 2.24  (1.86, 2.61) | 150.200  (101.751) | <.001 | 2.18  (1.29, 3.07) | 0.49 | -0.10  (-1.04, 0.85) |
| Digit Symbol Coding, n=658 | 52.060  (12.222) | 34.179  (9.843) | <.001 | -1.47  (-1.86, -1.09) | -- | -- | -- | -- | -- |
| STMS, n=677 | 35.962  (1.905) | 30.412  (2.797) | <.001 | -2.83  (-3.21, -2.46) | 24.333  (8.066) | 0.004 | -5.74  (-6.60, -4.87) | 0.02 | -1.55  (-2.49, -0.62) |
| **Neuroimaging Metrics**^1^ |  |  |  |  |  |  |  |  |  |
| Amyloid PET Meta-ROI SUVR, n=670 | 1.527  (0.341) | 1.780  (0.570) | 0.09 | 0.71  (0.37, 1.06) | 2.423  (0.721) | <.001 | 2.59  (1.83, 3.35) | 0.10 | 1.08  (0.23, 1.93) |
| Tau PET Meta-ROI SUVR, n=667 | 1.197  (0.098) | 1.288  (0.218) | <.001 | 0.84  (0.50, 1.19) | 1.881  (0.574) | <.001 | 6.08  (5.26, 6.89) | 0.04 | 1.97  (1.04, 2.89) |
| Tau PET EC-ROI SUVR, n=667 | 1.120  (0.130) | 1.296  (0.267) | <.001 | 1.26  (0.91, 1.61) | 1.672  (0.403) | <.001 | 4.09  (3.31, 4.87) | 0.09 | 1.29  (0.42, 2.15) |
| Hippocampal Volume z-score, n=680 | -0.283  (0.611) | -1.255  (1.097) | <.001 | -1.51  (-1.86, -1.16) | -1.477  (0.553) | <.001 | -1.96  (-2.71, -1.20) | 0.27 | -0.22  (-1.03, 0.60) |
| % WMH Volume, ln, n=665 | -0.680  (0.884) | -0.094  (1.060) | 0.41 | 0.66  (0.31, 1.00) | -0.193  (1.058) | 0.21 | 0.55  (-0.20, 1.29) | 0.64 | -0.09  (-0.91, 0.72) |

*Values are presented as Mean (Standard Deviation) unless otherwise noted.

^†^ All between-group comparisons are T-tests unless otherwise indicated.

^‡^ Chi-square

^§^ Fisher

^1^ Mayo Test Drive and Neuroimaging are independent of diagnosis (data not considered for consensus diagnosis).

^2^ Results remained significant when adjusting for age, sex, and education (all *p*’s ≤.003).

^3^ Results of in-person cognitive measures are considered for consensus diagnosis.

Note: ADRC = Alzheimer’s Disease Research Center; AVLT = Auditory Verbal Learning Test; CU = Cognitively Unimpaired; EC = entorhinal cortex; Mayo-PACC = Mayo Preclinical Alzheimer’s Disease Cognitive Composite; MCI = Mild Cognitive Impairment; MCSA=Mayo Clinic Study of Aging; MRI = Magnetic Resonance Imaging; MTD = Mayo Test Drive; MTD-SBCr = Mayo Test Drive Screening Battery Composite raw; MTD-SBCz = Mayo Test Drive Screening Battery Composite z; p = p-value; PET = Positron Emission Tomography; ROI = Region of Interest; SLS = Stricker Learning Span; STMS = Short Test of Mental Status; SUVR = Standard Uptake Volume Ratio; SYM = Symbols Test average correct item response time; SYM_aw_= Symbols Test accuracy-weighted average correct item response time; WMH = White Matter Hyperintensities.

*Supplemental Table 3.* Spearman Correlations between Neuroimaging Metrics and Cognitive Measures in the Full Sample

|  | **Amyloid PET**  **Meta-ROI SUVR** | | **Tau PET**  **Meta-ROI SUVR** | | **Tau PET**  **EC-ROI SUVR** | | **Hippocampal**  **Volume z-score** | | **% WMH**  **Volume, ln** | |
| --- | --- | --- | --- | --- | --- | --- | --- | --- | --- | --- |
| **Cognitive Measures** | rho | N | rho | N | rho | N | rho | N | rho | N |
| ***Remote*** |  |  |  |  |  |  |  |  |  |  |
| MTD-SBCr | -0.27*** | 666 | -0.24*** | 663 | -0.28*** | 663 | 0.26*** | 676 | -0.34*** | 661 |
| MTD-SBCz | -0.27*** | 666 | -0.24*** | 663 | -0.28*** | 663 | 0.25*** | 676 | -0.33*** | 661 |
| SLS Sum of Trials | -0.22*** | 667 | -0.23*** | 664 | -0.27*** | 664 | 0.22*** | 677 | -0.28*** | 662 |
| SYM | 0.28*** | 667 | 0.16 *** | 664 | 0.17*** | 664 | -0.26*** | 677 | 0.38*** | 662 |
| SYM_aw_ | -0.30*** | 667 | -0.17*** | 664 | -0.18*** | 664 | 0.26 *** | 677 | -0.38*** | 662 |
| ***In-Person*** |  |  |  |  |  |  |  |  |  |  |
| Mayo-PACC | -0.30*** | 649 | -0.14*** | 646 | -0.16*** | 646 | 0.28*** | 657 | -0.41*** | 642 |
| Global Cognition Z | -0.29*** | 617 | -0.14*** | 614 | -0.17*** | 614 | 0.31*** | 625 | -0.41*** | 611 |
| AVLT Sum of Trials | -0.22*** | 656 | -0.13*** | 653 | -0.17*** | 653 | 0.21*** | 664 | -0.29*** | 649 |
| Trail Making Test B | 0.31*** | 657 | 0.17*** | 654 | 0.17*** | 654 | -0.27*** | 665 | 0.44*** | 650 |
| Digit Symbol Coding | -0.29*** | 646 | -0.15*** | 643 | -0.13*** | 643 | 0.26*** | 654 | -0.40*** | 639 |
| STMS | -0.18*** | 664 | -0.13*** | 661 | -0.16*** | 661 | 0.24*** | 673 | -0.28*** | 658 |

*p<.05; **p<.01; ***p<.001

Note: AVLT = Auditory Verbal Learning Test; AVLT Sum of Trials = AVLT 1-5 total + Trial 6 + 30-minute delay; CU = Cognitively Unimpaired; Digit Symbol Coding = WAIS-R Digit Symbol Substitution Test; DM =Dementia; EC = entorhinal cortex; Global Cognition z = average z across all neuropsychological tests administered during an in-person visit; Mayo-PACC = Mayo Preclinical Alzheimer’s disease Cognitive Composite (average z of Auditory Verbal Learning Test sum of trials, animal fluency, and inversed Trails B); MCI = Mild Cognitive Impairment; MTD-SBCr = Mayo Test Drive Screening Battery Composite raw (Stricker Learning Span sum of trials + Symbols Test accuracy-weighted average correct items response time); MTD-SBCz = Mayo Test Drive Screening Battery Composite z; PET = Positron Emission Tomography; ROI = region of interest; SLS = Stricker Learning Span; SLS Sum of Trials = SLS 1-5 total + delay; STMS = Short Test of Mental Status, which is similar to the MMSE, is included for reference; SUVR = Standard Uptake Volume Ratio; SYM = Symbols Test average correct item response time; SYM_aw_= Symbols Test accuracy-weighted average correct item response time; Trail Making Test B = Trail Making Test B completion time; WMH = white matter hyperintensities.

*Supplemental Methods.* Detailed review of how to calculate the MTD-SBCr and SYM_aw_ variables.

mtdrawcompositev1 = MTD raw composite (MTD-SBCr)

symsumcorr = Symbols total correct across all 4 trials (0-48)

symsumcorr1to5scale = Symbols accuracy weighting to apply to SYM (1-5, as defined below)

symall4corrart = Symbols average correct items response time, seconds, across all 4 trials (SYM), range 0+

symall4corrartaccweighted = Accuracy-weighted Symbols average correct items response time (SYM_aw_)

slssumoftrials = Stricker Learning Span (SLS) sum of trials (range 0-108)

If one of the variables needed to do the calculation is missing, return as missing.

**Compute symsumcorr1to5scale as follows:**

If symsumcorr >= 47 then symsumcorr1to5scale = 5

If symsumcorr = 46 then symsumcorr1to5scale = 4.75

If symsumcorr = 45 then symsumcorr1to5scale = 4.5

If symsumcorr = 44 then symsumcorr1to5scale = 4.25

If symsumcorr = 43 then symsumcorr1to5scale = 4

If symsumcorr = 42 then symsumcorr1to5scale = 3

If symsumcorr < 42 AND symsumcorr > 34 then symsumcorr1to5scale = 2

If symsumcorr < 35 then symsumcorr1to5scale = 1

**Compute tenminussymall4corrartsec:** 10 - symall4corrartsec

**Compute symall4corrartaccweighted:**

- If tenminussymall4corrartsec < 0 then symall4corrartaccweighted = 0
- If tenminussymall4corrartsec > 0 then symall4corrartaccweighted = tenminussymall4corrartsec * symsumcorr1to5scale

**Compute mtdrawcompositev1** = slssumoftrials + symall4corrartaccweighted

**Composite Weighting Note:**

The range of the symall4corrartaccweighted is 0 – 50, in theory. A score of 50 is essentially impossible to attain, as this would require a 0 symbols average correct items response time (all timed responses must be > 0). The range of the MTD-SBCr is 0 – 158, in theory. Because of inclusion of symall4corrartaccweighted, this ceiling is theoretical and impossible to attain in practice. Because SLS sum of trials accounts for more of the total possible points (108/158), the MTD-SBCr is weighted more on SLS performance (68%) than Symbols (32%). The MTD-SBCz reported in the manuscript is similarly weighted more on SLS performance (75%) than Symbols (25%). MTD-SBCz is the average of SLS Maximum Learning Span z, SLS Trial 1-5 Total z, SLS Delay Total z, and SYM z (note SYM is inverted prior to averaging by multiplying by -1 such that higher scores reflect better performance).
